# Comparative analysis of Bricker versus Wallace ureteroenteric anastomosis and identification of predictors for postoperative ureteroenteric stricture

**DOI:** 10.1007/s00423-021-02413-4

**Published:** 2021-12-23

**Authors:** U Krafft, O Mahmoud, J Hess, J.P Radtke, A Panic, L Püllen, C Darr, C Kesch, T Szarvas, C Rehme, B.A Hadaschik, S Tschirdewahn

**Affiliations:** 1 Department of Urology, Urological Oncology and Pediatric Urology, University of Duisburg-Essen, University Hospital Essen, Essen, Germany; 2 Department of Urology, Semmelweis University, Budapest, Hungary

**Keywords:** Bladder cancer, Urinary diversion, Cystectomy, Bricker, Wallace

## Abstract

**Purpose:**

Ureteroenteric anastomosis after cystectomy is usually performed using the Bricker or Wallace technique. Deterioration of renal function is the most common long-term complication of urinary diversion (UD).

To improve surgical care and optimize long-term renal function, we compared the Bricker and Wallace anastomotic techniques and identified risk factors for ureteroenteric strictures (UES) in patients after cystectomy.

**Material and methods:**

Retrospective, monocentric analysis of 135 patients who underwent cystectomy with urinary diversion at the University Hospital Essen between January 2015 and June 2019. Pre- and postoperative renal function, relevant comorbidities, prior chemo- or radiotherapy, pathological findings, urinary diversion, postoperative complications, and ureteroenteric strictures (UES) were analyzed.

**Results:**

Of all 135 patients, 69 (51.1%) underwent Bricker anastomosis and 66 (48.9%) Wallace anastomosis. Bricker and Wallace groups included 134 and 132 renal units, respectively. At a median follow-up of 14 (6–58) months, 21 (15.5%) patients and 30 (11.27%) renal units developed UES. We observed 22 (16.6%) affected renal units in Wallace versus 8 (5.9%) in Bricker group (*p* < 0.001). A bilateral stricture was most common in Wallace group (69.2%) (*p* < 0.001). Previous chemotherapy and 90-day Clavien-Dindo grade ≥ III complications were independently associated with stricture formation, respectively (*OR* 9.74, 95% *CI* 2–46.2, *p* = 0.004; *OR* 4.01, 95% *CI* 1.36–11.82, *p* = 0.013).

**Conclusion:**

The results of this study show no significant difference in ureteroenteric anastomotic techniques with respect to UES development regarding individual patients but suggest a higher risk of bilateral UES formation in patients undergoing Wallace anastomosis. This is reflected in the increased UES rate under consideration of the individual renal units.

## Introduction

Radical cystectomy represents the standard treatment of muscle invasive bladder cancer (MIBC) and leads to long-term survival in 60% of MIBC patients [16, 28]. In addition, cystectomy can be performed due to non-urothelial malignancies and dysfunctional bladder or chronic pelvic pain syndrome. Performance status, renal function, and life expectancy and patients will have an influence on the choice of urinary diversion (UD). Several techniques and types of UD using different intestinal segments have been advocated, including ureterosigmoidostomy, urinary conduits, orthotopic bladder substitutes (OBS), and continent cutaneous diversions (CCD). The most common techniques to establish ureteroenteric anastomosis are the Bricker technique (separate, refluxing, end to side anastomosis) and the Wallace technique (conjoined, refluxing, end to end anastomosis). Anti-refluxing techniques have been abandoned due to higher ureteroenteric stricture (UES) rates [15, 17]. However, only few studies have compared the Bricker and Wallace technique, consequently, the choice of technique for ureteroenteric anastomosis is usually based on the surgeon’s preferences with limited evidence-based decision support [7, 11, 12, 21, 23, 32].

Regardless of the anastomotic technique, the most serious long-term complication of urinary diversion is the deterioration of renal function, mainly caused by UES, which affects 20–35% of patients [10, 19, 27]. Of particular interest was the question of bilateral affection of both upper urinary tracts under the conjoined Wallace anastomosis. In this analysis, we investigated the Bricker and Wallace techniques in terms of stricture formation and aimed to explore risk factors for UES.

## Materials and methods

A total of 201 patients who underwent cystectomy for any reason between January 2015 and June 2019 at the Department of Urology at Essen University Hospital were screened for inclusion. A total of 135 patients who received ileal conduit, orthotopic bladder substitute, and continent cutaneous diversion with a conclusive follow-up of at least 6 months were included in this retrospective monocentric analysis. The study was approved by the Ethics Committee of the University Hospital Essen (18–8349-BO) and performed according to the Declaration of Helsinki. A Bricker ureteroenteric anastomosis was performed in 69 patients, while the Wallace technique was applied in 66 patients.

We recorded the following preoperative patient data: age, gender, serum creatinine, body mass index (BMI), comorbidities, hydronephrosis, and indication for cystectomy. Physical condition was evaluated according to the classification system for physical condition of the American Society of Anesthesiologists (ASA). To evaluate the predictors of UES, we included previous treatments that may affect the outcome of anastomosis, including radiotherapy, chemotherapy, and abdominal surgery. Surgical data were recorded regarding the type of UD and the type of ureteroenteric anastomosis. Perioperative complications up to 90 days were analyzed. Only complications of grade ≥ III according to the Clavien-Dindo Classification system were recorded. The UES itself was considered an endpoint and was not registered as a complication (Table 1). The follow-up time was calculated for each patient. Patients with proven UES were identified and the time to UES diagnosis was calculated in addition to the treatment received. After postoperative ureteral stent removal, patients were discharged and continued to be followed by their outpatient physicians. When complications occurred, patients were referred to the hospital. In the absence of follow-up data, the outpatient physicians were consequently contacted after obtaining the patient’s written consent. UES was suspected in patients who showed symptoms indicating upper urinary tract obstruction or an increase in serum creatinine or in whom routine imaging revealed hydronephrosis. If hydronephrosis was confirmed by renal ultrasound, either a loopogram or a pouchogram was performed to rule out reflux. Initially a percutaneous nephrostomy was inserted to drain the kidneys. Subsequently, an antegrade contrast study including a Whitaker test was performed to diagnose a stricture. In addition, a computed tomography was conducted to rule out extrinsic ureteral compression, followed by either permanent nephrostomy, antegrade ureteral stent placement, or open revision. In unclear cases, a renal MAG3 (Mercaptuacetyltriglycine) scan was performed.

**Table 1:** Preoperative and operative patient’s characteristics

***p* value**	**Wallace group** (66 patients)	**Bricker group** (69 patients)	**Variables**
	132	134	Renal units, *n*
0.53	67.6 (9)	66.6 (10.8)	Age (years), mean (SD)
0.34	48 (72.7)	55 (79.7)	Male gender, *n* (%)
0.58	27 (4.4)	26.7 (5.4)	BMI (kg/m^2^), mean (SD)
0.03	1.2 (0.4)	1 (0.4)	Preoperative serum creatinine (mg ∕dl), mean (SD)
0.00	29 (43.9)	15 (21.7)	GFR (ml/min/1.73 m^2^) < 60, *n* (%)
0.26	11 (16.6)	7 (10.1)	Diabetes, *n* (%)
0.74	24 (36.3)	27 (39.1)	Vascular disease, *n* (%)
0.85	21(31.8)	23 (33.3)	Smoking history, *n* (%)
0.76	27 (40.9)	30 (43.9)	ASA score ≥ 3, *n* (%)
0.5	5 (7.6) 8 (12.1)	9 (13) 8 (11.6)	Preoperative hydronephrosis, *n* (%) Unilateral Bilateral
0.67	5 (7.5)	4 (5.8)	Preoperative chemotherapy, *n* (%)
0.26	6 (9.1)	3 (4.3)	History of pelvic radiotherapy, *n* (%)
0.7	9 (13.6)	11 (15.9)	History of abdominal surgery, *n* (%)
0.07	7 (10.9) 54 (81.8) 2 (3) 1 (1.5) 2 (3)	1 (1.4) 66 (95.7) 2 (2.9) 0 0	Indication for cystectomy, *n* (%) Benign disease Bladder cancer Prostate cancer Gynecologic cancer Colorectal cancer
0.38	54 (81.8) 10 (15.1) 2(3)	50 (72.4) 17 (24.6) 2 (2.9)	Diversion technique, *n* (%) Ileal conduit Orthotopic neobladder Continent cutaneous
0.9	2 (3)	2 (2.9)	Distant Metastasis, *n* (%)

### Surgical technique

Patients with bladder cancer underwent open radical cystectomy and lymphadenectomy. In the minority of patients (*n* = 8), the bladder was removed as part of pelvic exenteration for the treatment of advanced pelvic malignancies. In patients with non-oncologic disease, a simple cystectomy was performed. Urinary diversion was established as ileal conduit (*n* = 104), ileocaecal reservoir (*n* = 4), or neobladder in the Studer- or Hautmann configuration (*n* = 27).

Ureteroenteric anastomosis was routinely carried out using the Wallace technique until 2017 by surgeon group A; subsequently, the technique was changed to Bricker by a new group of surgeons (B). The change in anastomotic technique was accompanied by a change in the director of the department. Of note, however, operative and perioperative strategies did not change. Bowel preparation did not take place in either collective. In addition, the use of conduction anesthesia and opioid avoidance was applicated whenever possible. Intraoperative volume therapy was restricted to a goal of 1500 ml. Furthermore, there were no differences between the groups regarding stent removal (day 10–14). Similarly, there were no changes in perioperative nutrition and postoperative mobilization. The surgical technique of radical cystectomy did not differ between the groups except for the anastomotic technique of ureteroenteric anastomosis.

Both groups consisted of 3 surgeons each, of which the main surgeon in each group had over 10 year experience in performing urinary diversions. Surgeons of group A had already performed over 100 (CN) and over 300 (HR) procedures until 2015. Group B consisted of BH and ST. BH had performed over 200 and ST over 50 procedures until 2017. Each technique was performed exclusively by each group of surgeons, regardless of the type of diversion or patient characteristics. We enrolled patients only in the last 2 years of the Wallace era before switching to Bricker to ensure equal subgroups and symmetrical follow-up periods.

In Bricker anastomosis, we modified the classic technique by everting the mucosa of the ileal hole and fixing it to the surrounding ileal wall with 4 absorbable sutures [5]. Wallace I and II anastomoses were performed in the usual manner [31]. Regardless of the anastomotic technique, all ureteroenteric anastomoses were stented for 10 to 14 days.

### Statistical analysis

A comparison of means was performed using the Student *t*-test and a comparison of medians was conducted using the Mann–Whitney U test. Chi-square test was used to compare categorical variables. Logistic regression analysis was conducted to determine independent predictors of UES. *p*-values ≤ 0.05 indicated significance. All statistical analyses were performed using IBM SPSS Statistics for Windows, version 25 (IBM Corp., Armonk, NY, USA).

## Results

Among 201 cystectomies and UD, we identified 135 patients who met the selection criteria and completed the minimum follow-up period. The mean patients’ age was 67 years (± 10 years) and the majority of patients (76.2%) were male. The indication for cystectomy was urothelial carcinoma of the bladder, benign disease, and advanced non-urothelial pelvic malignancy in 89%, 6%, and 5%, respectively. The majority of patients underwent ileal conduit in 77%, followed by OBS in 20% and CCD in only 3% (Table 1). Of all 135 patients, 69 (51.1%) underwent Bricker anastomosis and 66 (48.9%) Wallace anastomosis. Four patients had a solitary kidney and received a Bricker anastomosis; therefore, the total numbers of renal units in the Bricker and Wallace groups were 134 and 132, respectively. There was no significant difference between both groups in terms of patient demographic and clinical characteristics, except for a lower preoperative renal function in the Wallace group in terms of higher serum creatinine (*p* = 0.03) and lower GFR (*p* < 0.001) in comparison to the Bricker group (Tables 1 and 2).

**Table 2:** Pathology results of cystectomy specimens

***p* value**	**Wallace group** (54 patients)	**Bricker group** (66 patients)	**Variable**
0.32	1 (1.8) 14 (25.9) 13 (24) 17 (31.4) 9 (16.6)	4 (6) 11 (16.6) 18 (27.2) 27 (40.9) 6 (9.09)	Tumor stage, *n* (%) T0 Ta, Tis, T1 T2 T3 T4
0.69	41 (75.9) 13 (24)	48 (72.7) 18 (27.2)	Nodal stage, *n* (%) Nx/N0 N1-3
0.5	2 (1–10)	1.5 (1–26)	No. positive lymph nodes, median (range)
0.73	50 (92.6) 4 (7.4)	62(93.9) 4 (6)	Histology, *n* (%) Urothelial carcinoma Non urothelial carcinoma

Perioperative outcome: within 3 months postoperatively, 33 (24.4%) patients developed Clavien-Dindo grade ≥ III complications. When comparing both cohorts, more major complications occurred in patients of the Wallace group, however, the difference was not significant (30.3% vs. 18.8%, *p* = 0.1). Significantly, more urinary leakage occurred in 8 (12.1%) patients of the Wallace group compared to 2 (2.9%) in the Bricker group (*p* = 0.04) (Table 3). Leakage was treated either conservatively or endoscopically in 5 patients, whereas surgical exploration was required in 5 patients of the Wallace group. Only one patient in the leakage group later developed UES.

**Table 3:** Postoperative parameters in Bricker and Wallace group comparison

***p* value**	**Wallace group** (66 patients)	**Bricker group** (69 patients)	**Variables**
0.19	16 (6–58)	14 (6–39)	Follow-up (months), median (range)
0.00	1.2 (0.4)	1 (0.2)	Postoperative serum creatinine (mg ∕dl), mean (SD)
0.18	20 (30.3)	13 (18.8)	90-day Clavien grade ≥ III, *n* (%)
0.04	8 (12.1)	2 (2.9)	Urinary leakage, *n* (%)
0.19	13 (19.7)	8 (11.6)	UES, *n* (%)
< 0.001	22/132 (16.6)	8/134 (5.9)	UES/renal units, *n* (%)
0.3	3 (1–38)	2.5 (1–16)	Median time to stricture, month (range)
0.00	4 (30.8) 0 9 (69.2)	6 (75) 2 (25) 0	Stricture laterality, *n* (%) Left Right Bilateral

UES development: after a median follow-up of 14 (6–58) months, 21 (15.5%) patients and 30 (11.3%) renal units developed UES. Median follow-up was similar between both groups (*p* = 0.1). Stricture was detected in 8 (11.6%) and 13 (19.7%) patients in the Bricker and Wallace groups, respectively (*p* = 0.19). However, the number of renal units affected was significantly higher with 22 (16.6%) in the Wallace group compared to only 8 (5.9%) in the Bricker group (*p* < 0.001). Regarding the side of affection, the left side was predominant in the Bricker group (75%) and no patient developed bilateral obstruction. In contrast, bilateral stricture was most common in the Wallace group (69.2%) (*p* < 0.001). All strictures occurred due to benign reasons except 1 patient in the Wallace group who developed tumor recurrence at the site of ureteroenteric anastomosis after 16 months (Table 3).

UES Management: out of 21 patients with stricture, 13 (59%) were treated with regular double-J-stenting, 4 patients (19%) were managed with open surgery, 3 (14.2%) patients did not receive any intervention due to impaired general condition, and 1 (4.7%) patient received a permanent nephrostomy.

Predictors of stricture development: when comparing stricture and non-stricture groups in terms of patients, pathological features and early postoperative complications, only the previous chemotherapy posed a risk for stricture formation. A total of 19% of patients in the stricture group had received chemotherapy for any reason prior to cystectomy, compared to only 4.4% in the non-stricture group (*p* = 0.01). On the other hand, smoking history was significantly higher in non-stricture group (36% versus 14.3%, *p* = 0.05) (Table 4).

**Table 4:** Comparison between stricture and non-stricture groups

*p* value	Non-UES group (114 patients)	UES group (21 patients)	Variable
0.99	66.6 (9.6)	69.9 (10)	Age (years), mean (SD)
0.9	87 (76.3)	16 (76.2)	Male gender, *n* (%)
0.2	26.7 (4.6)	28.2 (6.5)	BMI (kg/m^2^), mean (SD)
0.1	1.1 (0.4)	1.09 (0.18)	Preoperative serum creatinine (mg ∕dl), mean (SD)
0.88	15 (13.1)	3 (14.2)	Diabetes mellitus, *n* (%)
0.34	45 (39.4)	6 (28.5)	Cardiovascular disease, *n* (%)
0.05	41(35.9)	3 (14.2)	Smoking history, *n* (%)
0.36	50 (43.8)	7 (33.3)	ASA score ≥ 3, *n* (%)
0.06	37 (15.7)	9 (30)	Preoperative hydronephrosis/renal units, n (%)
0.01	5 (4.3)	4 (19)	Preoperative chemotherapy, *n* (%)
0.12	6 (5.2)	3 (14.2)	History of pelvic radiotherapy, *n* (%)
0.55	16 (14)	4 (19)	History of abdominal surgery, *n* (%)
0.69	87 (76.3) 24 (21) 3 (2.6)	17 (81) 3 (14.2) 1 (4.7)	Diversion technique, *n* (%) Ileal conduit Orthotopic neobladder Continent cutaneous
0.3	26 (22.8)	7 (33.3)	90 day-Clavian grade ≥ III, *n* (%)
0.61	9 (7.9)	1 (4.7)	Urinary leakage, *n* (%)
0.89	56 (49.1)	10 (47.6)	Local advanced dis., *n* (%)
0.27	30 (26.3)	8 (38.1)	Node positive dis., *n* (%)

We adjusted the preoperative chemotherapy in the regression analysis with the other known risk factors for stricture formation, which included BMI, anastomotic technique, preoperative chemotherapy, and early Clavien-Dindo grade ≥ III complications (Table 5). The administration of preoperative chemotherapy was an independent predictor for stricture formation (*OR* 9.74, 95% *CI* 2–46.2, *p* = 0.004). In addition, the occurrence of 90 day-Clavien-Dindo grade ≥ III complications in multivariate analysis was associated with a high risk for stricture formation (*OR* 4.01, 95% *CI* 1.36–11.82, *p* = 0.012). The type of ureteroenteric anastomosis (Bricker versus Wallace) was not associated with risk of UES development.

**Table 5:** Multivariate analysis for predictors of UES

*P* value	Adjusted *OR* (95% *CI*)	Variable
0.304	1.05 (0.95–1.15)	BMI
0.004	9.74 (2–46.2)	preoperative chemotherapy
0.012	4.01 (1.36–11.82)	90-day Clavian grade ≥ III
0.38	1.56 (0.56–4.31)	Anastomotic technique (Wallace vs Bricker)

## Discussion

Renal dysfunction is the most serious long-term complication related to UD. Among other factors such as patient age, chronic hypertension, diabetes, preoperative renal function, and diversion-related factors, UES represents the most common and critical risk factor for deterioration of renal function [10; 13; 19]. The incidence of UES varies from 2.6 to 13% in the literature [1–3, 14, 17, 26, 29, 33]. The median time of development of an UES is between 7 and 12 months after UD and usually occurs in the first 2 postoperative years [20, 21, 24, 26, 27, 30, 33].

We encountered a slightly higher incidence of 15.5% in our patients. However, comparison of stricture rates between published studies is difficult because all of these studies are retrospective and lack standardized follow-up protocols. On closer attention to some large series that reported lower stricture rates, we found out that they focused only on benign UES [1, 2, 14, 25, 26, 33], some of them did not include all cystectomies in the analysis, and excluded patients with preoperative risk factors for stricture development, such as radiotherapy [14].

In addition, some studies included patients with less than 6-month follow-up, which is considered a short time for evaluation [2, 3, 14, 26, 33]. In our comparative study, we tried to avoid underestimation of strictures to ensure that we included all the patients regardless of their initial characteristics and analyzed the stricture rate as either benign or malignant; furthermore, we included only patients who had completed at least 6-month follow-up.

We analyzed risk factors for the development of UES and found that preoperative chemotherapy was associated with a nearly ninefold higher risk of developing a stricture for this cohort. However, only 9 patients received chemotherapy before surgery for any reason. None of the previous large studies observed this association. Yang et al. examined risk factors for UES in 2.285 patients. A total of 613 (27%) patients received perioperative chemotherapy, and it was not a predisposing factor for UES [33]. In another large study, 580 patients (20%) received neoadjuvant chemotherapy and it was a predictor of stricture only in univariate analysis (*HR* 1.56, 95% *CI* 1.03–2.36, *p* = 0.036) [2]. In addition to the well-investigated long-term cardiovascular damage caused by platin-containing chemotherapy, a directly mediated acute endothelial damage was recently suspected. Furthermore, particularly, Gemcitabine as well as Cisplatin is highly suspected to cause a thrombotic microangiopathy. Therefore, cytotoxic therapy may play a role in decreasing oxygen supply and thus cause fibrotic remodeling at the anastomotic site [9]. Further large studies are needed on this topic.

In colorectal and transplantation surgery, the perfusion of kidney transplants and enteroanastomoses can be tested intraoperatively using ICG angiography. Recently, we introduced this technique study-based to reduce the risk of scarring or leakage with consecutive UES [4, 22].

Postoperative complications may predispose to UES development, such as urinary or intestinal leakage, abdominal abscess, and UTI. These factors can trigger an inflammatory process at the anastomotic site and predispose to scar and UES formation. Thus, similar to some previous reports, overall occurrence of major complications in our study was an independent risk factor for UES [25, 33].

Urinary leakage is considered a risk factor for the development of UES in 2 of the studies mentioned above [1; 25]. In our cohort, urinary leakage rarely originated from the ureteroenteric anastomosis but from the neobladder or pouch itself, which may explain the lack of stricture development in these patients. Similarly, high BMI is a well-studied risk factor for the development of UES [1, 2, 23, 33]. Probably due to the sample size of our work, we did not find a compelling association in this context.

The only published meta-analysis comparing Wallace and Bricker techniques was based on 4 retrospective studies with rather small cohorts and showed a comparable stricture rate for both techniques (Bricker: 2.9% vs. Wallace: 1.9%, *p* = 0.57) [7, 8, 12, 21, 23]. In contrast, we found a higher UES rate for the Wallace group during similar follow up periods, as 9 patients in the Wallace group developed bilateral UES, whereas none in the Bricker group experienced bilateral disease.

Our results are consistent with the largest study in this concern. Evangelidis et al. compared both groups in terms of the number of renal units involved. They considered Wallace anastomosis as one unit and Bricker anastomosis as 2 units. UES was found in 3 (1.85%) versus 5 (4.46%) units in the Bricker and Wallace groups, respectively (*p* = 0.208). Consequently, considering the affected renal units, 3 and 8, UES in the Bricker and Wallace group were observed, which leads to a significant difference [12]. On the contrary, 2 of the aforementioned publications found higher stricture rates for Bricker anastomosis. Kouba et al. reported the absence of strictures in Wallace compared to 3.7% in Bricker, although this group was characterized by significantly increased BMI, which is a well-investigated risk factor for stricture formation [21]. In addition, the most recent study found a stricture rate in Bricker and Wallace of 25.3% and 7.7%, respectively (*p* = 0.024), possibly resulting from the unequal length of follow-up in the Bricker group in this study [6]. In our opinion, the main disadvantage of the Wallace technique is the conversion of 2 renal units into one; therefore, any pathologic lesion at the site of ureteroenteric anastomosis may affect both renal units. Limitations of our study include the retrospective character with a limited sample size. Furthermore, among the limitations, the influence of individual surgeon teams must be mentioned. Although both teams have a very similar experience level, finally, an influence cannot be excluded. Interestingly, there were no changes in the perioperative protocols as specified above.

Strikingly, our collective includes 25 patients who underwent radical cystectomy for non-muscle invasive urothelial carcinoma of the bladder. In our department, early cystectomy is regularly offered because of the assumption of at least 20% of understaged patients after TUR-BT. Furthermore, a survival benefit for early versus deferred cystectomy was reported [18].

## Conclusion

The results of this study show no significant difference in ureteroenteric anastomotic techniques with respect to UES development regarding individual patients, but suggest a higher risk of bilateral UES formation in patients undergoing Wallace anastomosis. This is reflected in the increased UES rate under consideration of the individual renal units. Despite the limitation of sample size, previous chemotherapy represented a potential risk factor for UES development in this analysis.

## Data Availability

All data were analyzed anonymously in accordance with the data protection guidelines of our facility.
